# 2030: “Understandable to the subject”: Plain language IRB informed consents

**DOI:** 10.1017/cts.2017.187

**Published:** 2018-05-10

**Authors:** Tina Moore, Laura P. James, Jennifer Holland, Edith Paal, Kristie Hadden

**Affiliations:** Translational Research Institute, University of Arkansas, Fayetteville, AR, USA

## Abstract

OBJECTIVES/SPECIFIC AIMS: Develop a plain language informed consent template that met IRB and regulatory requirements. Evaluate the effectiveness of the template at improving the readability of informed consents. Field test the informed consent with low health literacy. METHODS/STUDY POPULATION: We conducted a retrospective analysis of over 200 UAMS IRB approved, investigator initiated informed consents from 2013 to 2015 to determine the readability before intervention. The mean grade level readabilities were derived from the results of 3 readability formulas (Flesch-Kincaid, SMOG, and Fry) using open-source readability tools. A plain language informed consent template that meets IRB and regulatory requirements was developed, adhering to health literacy best practices for written communication. The template was made available to investigators as an optional resource, and IRB committees were trained on use of the template. In addition, a focus group will be conducted to qualitatively assess understandability of the template with study participants identified as having inadequate health literacy. Data analysis will include readability assessment of IRB approved informed consents post intervention with and without use of the plain language template, as well as qualitative feedback from focus group participants. RESULTS/ANTICIPATED RESULTS: The retrospective analysis revealed a mean readability of 10th grade for IRB approved informed consents from 2013 to 2015 (n=217). The readability of the developed plain language template was 5th grade. Preliminary post-intervention results show adoption of the template by investigators (n=16) resulted in informed consents with a mean readability of 7th grade (range 6–9th grade), compared to a mean of 10th grade (range 7–11th grade) for the comparator (“no adoption” group, n=24). Data collection will continue through May 2017. The focus group is forthcoming and results will be included in the poster. DISCUSSION/SIGNIFICANCE OF IMPACT: Low health literacy is common in individuals with healthcare disparities and can limit their participation in clinical research. Few studies have examined interventions to address this barrier to research. Preliminary results of this study support the utilization of a plain language informed consent template in investigator-initiated research. Moreover, this study demonstrates the importance of stakeholder engagement among CTSA leadership, health literacy experts, the institutional review board, investigators, and research subjects in the development and testing of this intervention to make informed consents “understandable to the subject” while containing all required elements.

